# The Client Network Audit: Assessing Shared Knowledge of a Client’s Social Network Among Juvenile Probation Officers

**DOI:** 10.3390/bs16040614

**Published:** 2026-04-20

**Authors:** Jacob T. N. Young

**Affiliations:** School of Criminology and Criminal Justice, Arizona State University, 411. N Central Ave., Phoenix, AZ 85004, USA; jacob.young.1@asu.edu

**Keywords:** group audit, youth violence, social network analysis, cognitive social structures

## Abstract

This article presents findings from a pilot that tested a novel “client network audit” approach, designed to enhance supervision by mapping social networks through structured input from frontline practitioners. Adapting the group audit methodology for collecting network information that is used extensively in gang violence interventions, this project measured cognitive network data from two probation officers and a community-based partner to examine areas of consensus and divergence in perceptions of influential relationships in a client’s life. A focus group conducted with participants after the study revealed several themes, including the utility of identifying hidden risks and opportunities for intervention and enhancing multi-agency coordination. This exploratory study finds that, while there are key areas of overlap in these perceptions, there are substantial gaps, indicating that individuals possess unique information about social relationships that is unknown to other respondents. As jurisdictions seek innovative strategies to improve interventions for youth entrenched in high-harm networks, this model offers a potentially promising pathway.

## 1. Introduction

Network science demonstrates that, for many types of behavior, ranging from health outcomes (e.g., Centola, 2010) to online hate speech (Thomas & Wahedi, 2023), understanding the connections between actors in these settings can enhance the design and effectiveness of interventions designed to alter behavior. *Network interventions* involve the use of social network information to facilitate behavior change by employing four strategies: identifying influential individuals, targeting specific segments or modules of the network, diffusing or inducing diffusion of messages, or altering network links (see Valente, 2012, for a review; see also Robins et al., 2023). However, a key insight from work in this area is that effective interventions depend on a critical feature: knowledge of the network.

A key fact in criminology is that many juvenile offenses occur in groups (Klymentiev et al., 2025). As such, network interventions have been used in multiple settings involving criminal justice outcomes, from substance use (e.g., Osgood et al., 2013) to gang violence (Sierra-Arevalo & Papachristos, 2017), to reduce problematic behavior. While most criminal justice research incorporating network information to inform interventions is gleaned from official data (e.g., co-arrest information, court transcripts, school rosters), the limitations of these sources have been extensively documented (see Bright et al., 2021). In contrast, the pioneering work of D. M. Kennedy et al. (1997) recognized that “some of the richest information for describing public safety problems and driving problem-solving efforts simply is not available from any official data systems” (D. M. Kennedy et al., 1997, p. 226). Rather, D. M. Kennedy et al. (1997) argued that through their routines, criminal justice practitioners acquire *experiential knowledge* regarding the problems they work with daily. The insight that information about social networks is embedded in the minds of criminal justice practitioners and representatives of community-based organizations (CBOs) was a key insight, leading to the development of the “group audit” methodology, which has been central for reconceptualizing the causes of and developing effective interventions for gang violence (see Sierra-Arevalo & Papachristos, 2017, for a review).

While the group audit method has been productive for addressing gang violence, there are two key gaps that deserve greater attention. First, the group audit method has been limited in its application and has not been used in criminal justice settings where similar problems confront actors seeking to serve their clients. For example, probation officers face the same problem when seeking ways to serve their clients: individuals under supervision are embedded in social relationships that influence their behavior. While existing research using the group audit method has focused on police- and gang-related violence, the approach may have wider application to practitioners in the criminal justice system (e.g., to probation officers and CBOs). The potentially fruitful application of the method stems from the possibility that practitioners’ experiential knowledge from sustained community contact is a largely untapped resource for understanding and addressing youth offending.

The second issue stems from a limitation of the group audit method. Past work employing the method often treats the network as an ontologically measurable entity, as if there is a “true” network that can be measured (see Sosa & Rodríguez, 2021, p. 86). But the network that is measured and employed in an intervention is a product of individuals’ beliefs about social relationships. These “cognitive social structures” have important areas of both overlap and divergence. To date, these have not been quantified or incorporated into a research design. Identification and inclusion of these concepts provide a potentially valuable extension to the group audit method.

This study contributes to both gaps by describing a pilot project that adapted the group audit method to address the needs of probation officers. The project developed a “client network audit” with two probation officers and one representative of a CBO, mapping a single client’s social relationships. The goal was to collect social network data on a client and assess how the client’s social environment might shape behavior and support supervision efforts. More broadly, the pilot aimed to test a scalable data collection approach that could help the participating probation agency to reduce re-offending and improve public safety. In the following sections, the history of the group audit method is discussed, followed by an overview of the literature on cognitive social structures as it pertains to the current study. The remaining sections describe the methodology and the findings of this exploratory study. The manuscript ends with a discussion focused on extending the approach, including the limitations of the current pilot and additional considerations for future research.

### 1.1. Group Audit

The group audit method was developed by D. M. Kennedy et al. (1997) as part of a project seeking to reduce gun violence in Boston. After multiple conversations and structured interviews with police officers, probation officers, and community members, the researchers gained an understanding of the nature of the problem they were trying to solve: that violence in communities was concentrated among a small set of individuals who were repeatedly involved in crime. The implication was that interventions should be designed in such a way that they identify specific individuals who are most actively involved in violence (as opposed to uniformly applying an intervention to everyone), a key approach of network interventions (Valente, 2012). To identify who should be involved in the intervention, D. M. Kennedy et al. (1997) developed the group audit method, whereby social network information is collected from practitioners and community members through multiple meetings in which they discuss gang geographic boundaries and inter-group conflicts to identify the turf boundaries and antagonisms (as well as alliances) between the gangs. This process produces a “consensus” network that is used to aid in intervention design.

The group audit method is built on the logic that “practitioners and community members can make powerful contributions to identifying and understanding crime problems” because the “experience, observations, local knowledge, and historical perspective of working police officers and others with routine contact with offenders, communities and criminal organizations may represent an important underutilized resource for describing, understanding, and crafting interventions aimed at crime problems” (D. M. Kennedy et al., 1997, p. 226). These “experiential assets” were a key source of data that informed the intervention. As D. M. Kennedy et al. (1997, p. 236) state, “We thus faced two important questions: (1) Which gangs would be the most efficient to target if police agencies wanted to disrupt key sources of conflict? (2) How could we best diffuse the deterrent message across Boston’s gang landscape?” This “focused deterrence” approach thus depended on knowing the network. The group audit has been a key data collection component of focused deterrence strategies and has been examined in multiple cities, including Chicago (Papachristos & Kirk, 2015), Philadelphia (Hyatt et al., 2021), Newark (Mcgloin, 2005), and New Haven (Sierra-Arevalo et al., 2016).

A key insight from the research on network interventions designed to reduce gang violence is that valid knowledge of the network is critical. While the group audit method has been limited in its application (i.e., gang violence), the basic insight that practitioners and community members possess valuable information for informing the design of interventions extends beyond gangs to additional settings where similar problems regarding the collection of data affect criminal justice actors. Perhaps one of the most fruitful areas of application is for probation officers. Probation officers face the same problems and knowledge gaps as they seek ways to serve their clients. Individuals under supervision are embedded in social relationships that influence their behavior, and knowledge of these relationships may be an important source of information for guiding supervision. The same is true for individuals working in CBOs whose mission is to reduce problem behavior in the areas they serve. These individuals have a deep knowledge base of social relationships in the community (see Hureau & Papachristos, 2025), and such knowledge might be of value for delivering services to clients on probation. Probation officers and members of CBOs most likely possess experiential assets that could be of value, representing a largely untapped resource for understanding and addressing youth offending. Put simply, these areas of study and practice may benefit from adapting and implementing the group audit method.

### 1.2. Cognitive Social Structures

Though the group audit method has shown promise for gaining insight into the social relational structures that might be relevant to a network intervention, research employing this tool has largely ignored some basic issues regarding the measurement of the network. As discussed above, the group audit method collects social network information from practitioners and community members, creating a “consensus” network based on these discussions. This network, a product of information from multiple individuals, is then used in designing and implementing the intervention.

However, this procedure does not consider the fact that the network used for the intervention is a product of individuals’ *beliefs* about social relationships. While this data structure captures the overlap among practitioners and community members, it fails to quantify the extent of consensus or to identify areas where there are divergent beliefs. These concepts (i.e., consensus and divergence) represent important properties of the information or knowledge that is then used to design network interventions.

Thus, a key gap in the group audit method literature is the ability to quantitatively examine the extent of consensus. This point was recognized by D. M. Kennedy et al. (1997, p. 251): “…we lack a literature on police practitioner information, knowledge, and perception equivalent to that on reported crime and calls for service, we have considerably less outside theory and analysis to bring to bear on interpreting our results…It seems to us, in fact, that these are literatures that badly need creating.” (p. 251). This study extends the group audit method by integrating work on cognitive social structures to attend to the issues raised by D. M. Kennedy et al. (1997) to develop a foundation for developing literature on this topic.

There is a large body of literature on how individuals construct social maps in their minds as they experience the world (see Smith et al., 2020 for a review). *Cognitive social structures* are cognitive representations of social networks (Krackhardt, 1987; see Brands, 2013 for a review) and describe patterns of interactions as perceived by individuals. Cognitive social structure data are collected by asking individuals to report their perceptions of relationships. For example, does Jim think Susan and Tim are friends? Does Susan think Jim and Tim are friends? Does Tim think Susan and Jim are friends? These data represent individuals’ beliefs about friendships and allow for the quantification of overlap/consensus, or lack thereof, among individuals in their beliefs.

The literature on cognitive social structures is directly applicable to the group audit method, as this approach to data collection is essentially seeking to elicit these maps from individuals and examine their concordance across multiple actors. However, an important distinction from work on cognitive social structures is that the group audit method is primarily focused on conducting social network analysis on the data object gleaned through the audit process. Cognitive social structure studies are more concerned with individuals’ perceptions of that structure. While knowledge of the network is important for network interventions, understanding the properties of how that knowledge is organized (“is there consensus?”) is equally important. Cognitive social structures provide an avenue for assessing convergent and divergent knowledge among a set of individuals regarding the social space they are seeking to understand.

These differences in knowledge were apparent to D. M. Kennedy et al. (1997, p. 229): “These different groups of practitioners were focused on the same basic issue…, their sources of information overlapped to some degree. However, the groups worked from bases of experience that were meaningfully different” (p. 229). These “meaningfully different” experiences are potentially useful for understanding the environment in which network interventions occur and broaden the set of questions that can be examined. For example, do individuals differ in their beliefs about a network because they have different positions in the network? A probation officer’s knowledge of a client’s network may be limited to direct family and/or other justice-involved individuals. Alternatively, a CBO representative may only observe family members and friends who live in the same community as the client. In other words, individuals may have access to different knowledge. Documenting and measuring this divergence may prove insightful for designing interventions.

Finally, a strength of reframing the group audit method through the lens of cognitive social structures is that it does not assume that there is some “true” network that exists; that is, there is some observable network that exists, in an ontological sense, and we just need to objectively measure it. On the contrary, “networks are purely a consequence of the behavior of individuals when we force them to express their (spatial) understanding in terms of a network instrument” (Sosa & Rodríguez, 2021, p. 86). Rather, this approach assumes that “perceptions provide valuable information about the network and through them, multiple notions of the network exist” (Aguiar & Ugander, 2024, p. 202). In sum, focusing on cognitive social structures represents a first step in understanding the structure of knowledge of the network that would occur prior to designing a network intervention.

## 2. Materials and Methods

The “client network audit” pilot sought to measure the “experiential knowledge” of probation officers and members of CBOs. The instrument was designed to map the connections among those known to be involved in illegal activity and their potential connectivity with others in a joint enterprise to provide a means to assess how the social context in which their clients live may influence their behavior and aid probation officers with client supervision. The overarching goal was to explore a novel data collection strategy to assist the participating probation agency in reducing re-offending and enhancing public safety.

The pilot research proposal was submitted to the research team for the probation agency in February 2024 and was approved in November 2024. The lead researcher received Institutional Review Board (IRB) approval to conduct the research in February 2024 from the associated university. In mid-October 2024, a site for the pilot was identified, and case files were reviewed by the lead representative of the probation agency to identify one client who was a good candidate for the pilot. The pilot was restricted to a single client to assess the feasibility of the approach without excessively burdening the probation agency. Two probation officers and one CBO representative were selected to participate in the pilot (henceforth referred to as respondents). Two weeks prior to data collection, the respondents were briefed on the pilot and were asked to think about the social relationships of their clients to prepare them for data collection. This was done so that respondents had an opportunity to think about these networks as they went about their work. On the day of data collection, the lead researcher met in person with the three respondents and representatives of the probation agency. Respondents were briefed again on the aims of the project and the protocol for data collection. After the meeting, the respondents were led to separate locations to begin data collection.

**Figure 1:**
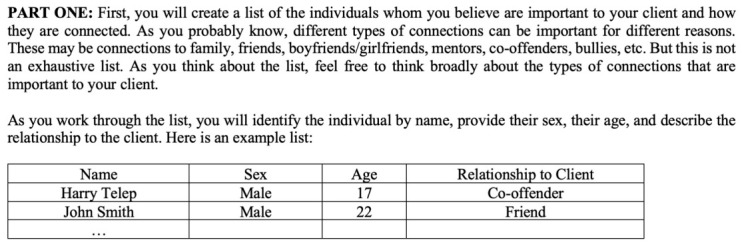
Section 1 of Instrument.

**Figure 2:**
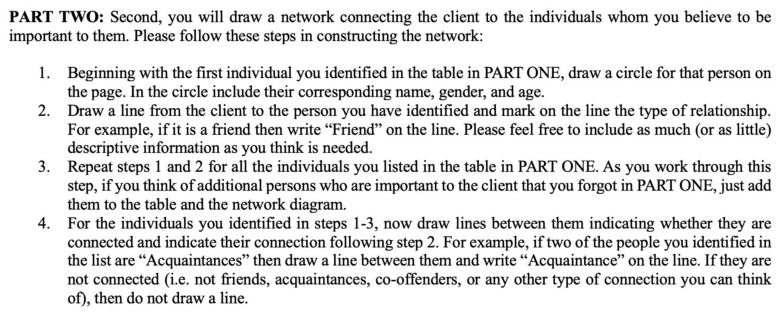
Section 2 of Instrument.

**Figure 3:**
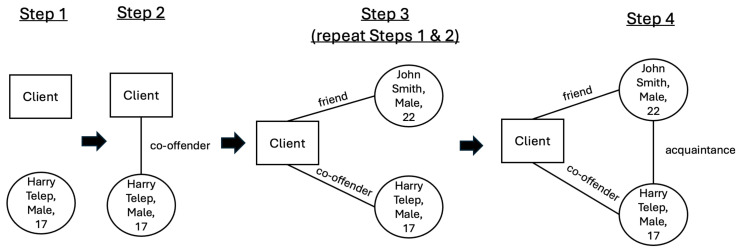
Visual Aid provided in Instrument.

During data collection, the respondents independently created network diagrams based on a two-part process. In the first part, individuals were asked to create a list of individuals whom they believed were important to the client and to identify the nature of the connection (e.g., family member, friend or acquaintance, co-offender). This section of the instrument is shown below in Figure 1:

In network terminology, the client is called the ego, and the people identified as influential are called alters. The first part, therefore, establishes the ego–alter connections. In the second part, individuals created a network connecting the focal client (ego) to the individuals (alters) whom they believed to be important. After drawing these connections, respondents were then asked to draw connections between the alters and indicate the nature of their connection. This step, therefore, creates the alter–alter connections. This section of the instrument is shown below in Figure 2:

As the instructions were new to the participants, a figure illustrating the process was provided. Figure 3 below illustrates the data collection sequence and was provided to the respondents as a visual aid:

Respondents completed the instrument with a member of the probation agency in case they had questions and/or needed clarification. After completing and reviewing the diagram, each network was then anonymized by a member of the probation agency and redrawn. The anonymized networks replaced all real names with unique codes. The anonymized physical maps were then mailed to the lead researcher, who used them to prepare electronic versions for analysis.

## 3. Results

### 3.1. Exploring Consensus and Divergence in Cognitive Social Structures

**Figure 4:**
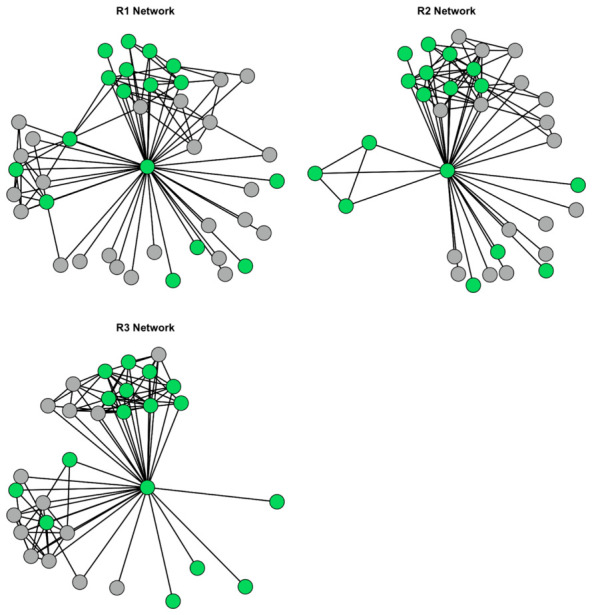
Cognitive Social Structures for each Respondent (green nodes are individuals identified across all three respondents).

Each respondent drew a separate network representing their beliefs about which relationships were important in the client’s life, providing three network diagrams for examining consensus and divergence in these beliefs. The belief networks are referred to as R1’s network, R2’s network, and R3’s network. In each network, the client who was the focus of the pilot received the unique ID “E” (short for ego). The network for each respondent is shown in Figure 4:

Respondents were allowed to freely identify all alters in the client’s (i.e., ego’s) network. The first step in the analysis is to explore the agreement on alters between each of the respondents (this is the ego–alter network). In total, 59 individuals were identified across all three networks. Of these 59 alters, only 16 were identified in all three networks (i.e., occurred in R1, R2, and R3’s diagrams). This means that of all the individuals identified, only 27% exhibited consensus ego–alter ties between all three respondents. Between R1 and R2, there were 22 consensus ego–alter ties (37%), while between R1 and R3, there were 22 consensus ego–alter ties (37%). Between R2 and R3, there were 17 consensus ego–alter ties (29%).

The next units of analysis are the ties from alter to alter (the alter–alter network), as identified by each respondent. Here, the question regards the extent of overlap between respondents in terms of the ties they perceive between alters. This agreement can be examined using the Jaccard index, where *A* and *B* are networks, and the quotient represents the proportion of ties from alter to alter in network *A* that appear in network *B*. The Jaccard indices for these data are 0.13 for R1 and R2, 0.13 for R1 and R3, and 0.04 for R2 and R3. In other words, there is very little consensus across the respondents in terms of the alter–alter networks.

As a final step, the ego–alter and alter–alter nominations can be examined together using the same measure to get an overall sense of how much consensus there is across respondents. The Jaccard indices for these data are 0.24 for R1 and R2, 0.23 for R1 and R3, and 0.13 for R2 and R3. Adding the ego–alter ties shows that much of the consensus among the respondents is due to the ego’s ties to alters. This indicates that, while there is some overlap between the respondents, there is considerable variation between whom they report to be influential in the client’s life, as is particularly the case when alter–alter ties are examined.

**Figure 5:**
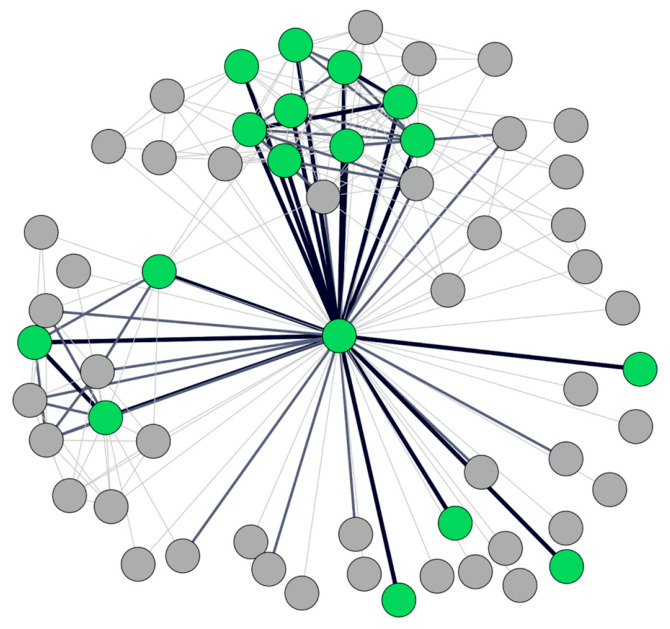
Composite Network for Cognitive Social Structures.

The overlap (or lack thereof) can be visualized by creating a weighted graph by “stacking” each individual network from the respondents. This *composite* network, where thicker edges indicate more overlap in ties reported across the respondents, is shown in Figure 5. In this plot, green nodes are those whom all three respondents identified in their networks (*n* = 16), and gray nodes are those whom only one or two respondents identified (*n* = 43).

**Figure 6:**
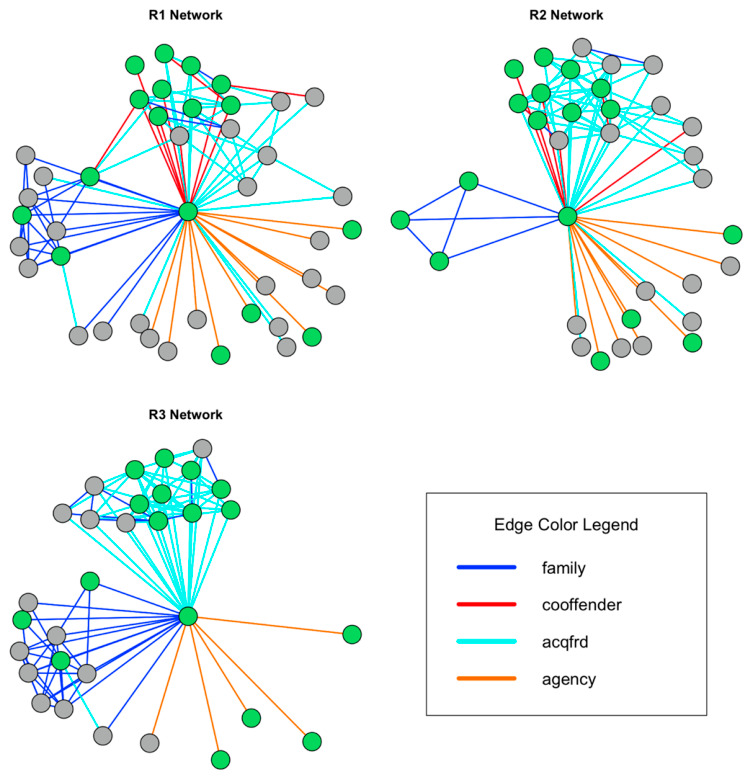
Cognitive Social Structures by Edge Type for each Respondent (edges colored by type).

In addition to identifying ego–alter and alter–alter ties, respondents also indicated the type of relationship that characterized each tie. To visualize these differences, Figure 6 shows the respondent networks, where connections between individuals are colored based on the type of tie identified by the respondent and reclassified as either *family*, *acquaintance/friend*, *co-offender*, or *agency*. Individuals who were not identified by a specific respondent are excluded from that respondent’s network to aid with visualization. Looking across the networks, the lack of overlap is made clear by the inconsistency of certain individuals in the plots. As with Figure 5, the color of the individual corresponds to whether the individual was nominated across all three diagrams.

In addition to identifying unique individuals who are influential to the client, another important finding shown in the plot above is that respondents differed in the extent to which they identified types of connections. Table 1 below shows the percentage of connections that are represented by each type of tie for each respondent:

The table illustrates several important features. For R1, family and acquaintance/friend ties make up the bulk of the network, comprising 75.86% of the ties. For R2, acquaintance/friend ties make up the bulk of the network, comprising 68.29% of the network. In contrast to both R1 and R3, for R2, family ties represent only a small fraction (9.76%) of the ties. For R3, family and acquaintance/friend ties make up the bulk of the network, comprising 94.84% of the network. For R1 and R2, co-offender ties make up 11.49% and 9.76% of the network, respectively, whereas for R3, co-offender ties make up 0% of the network.

Again, employing the Jaccard index, we can examine overlap across the networks (where the units of analysis are ego–alter ties and alter–alter ties combined). For *family* ties, the index scores are 0.15 for R1 and R2, 0.27 for R1 and R3, and 0.08 for R2 and R3, offering an average score of 0.17. For *acquaintance/friend* ties, the index scores are 0.10 for R1 and R2, 0.09 for R1 and R3, and 0.09 for R2 and R3, offering an average score of 0.09. Finally, for *agency* ties, the index scores are 0.50 for R1 and R2, 0.45 for R1 and R3, and 0.36 for R2 and R3, offering an average score of 0.43. These calculations indicate that there are key differences in the overlap for the different types of relationships, with agency ties showing much more overlap compared to family or acquaintance/friend ties. These comparisons help reveal where there are gaps between respondents regarding their cognitive maps of a client’s social setting.

**Figure 7:**
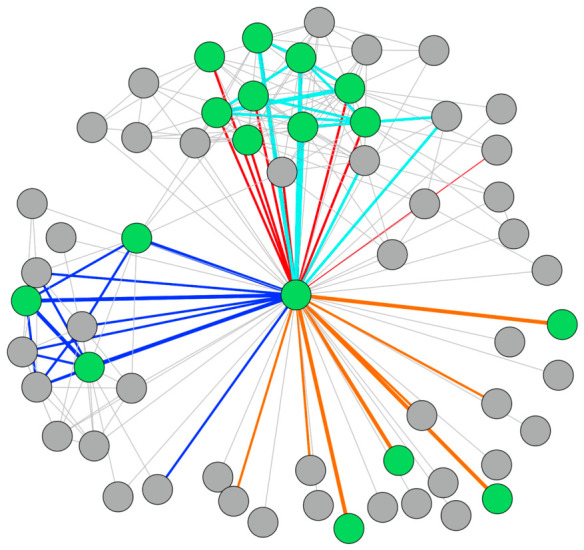
Composite Network for Cognitive Social Structures by Edge Type.

As was done above, a composite network can be created by “stacking” each of the networks to create a weighted network. Figure 7 shows the composite network, where the thickest lines are those connections identified by all three respondents as the same type. The ties in which only two respondents provided the same designation are colored, but the line widths are thinner. The gray connections are those where either two respondents identified the tie but indicated a separate type (i.e., inconsistent naming) or only one respondent provided that tie. In other words, the other respondents did not report a connection for that individual.

### 3.2. Focus Group Feedback

After the pilot, a focus group was conducted with participants to gather reflections on the utility, feasibility, and potential application of network data in the context of client supervision. The focus group was conducted via Zoom with the participants, the lead researcher, and two representatives of the probation agency. For the focus group, the lead researcher created a web-based application using the pilot data to assist with the visualization of the networks. The “Probation Pilot Tool” (available at https://jacobtnyoung.shinyapps.io/probation-pilot-app/, accessed on 27 January 2026) allows the user to select each of the networks from the pilot, display them, and adjust various visual parameters (e.g., the size of the individuals, transparency of connections, size of lines). The application also allows the user to upload a list of names that correspond to the unique IDs shown in the plot. This allows the respondent to reference the actual names of those identified during data collection but maintains anonymity for the researcher (or other parties who might be sharing the anonymized data).

Before beginning the discussion, participants were reminded of the pilot project’s goals and the data collection process. The instrument used to collect the network data was reviewed, and the participants discussed how the information was visualized in the resulting network plots. The group then worked through the Probation Pilot Tool and went through several questions. Thematic analysis (Braun & Clarke, 2006) was used to identify themes. As the focus group was not a separate data collection phase, the discussion was not recorded. Coding was conducted during the discussion, noting key terms that were used by the participants during the process. After the focus group ended, the researcher compiled these terms and phrases into themes. These results were then reviewed by the two representatives of the probation agency to corroborate the interpretation of the salient themes of the focus group. Below, each question and the general themes of the feedback are provided.

Theme 1: “Reflections on Network Content”. This came from the following question: “*The goal of this pilot was to identify relationships in a client’s life that are important. In looking at the networks, what stands out to you?*” Respondents generally agreed that the tool was useful. One participant highlighted that the process of first writing out the connections for the client, drawing the maps, and finally transitioning to the network visualization was particularly effective for illustrating who is connected to whom. The visual format helped make the significance of certain relationships more immediately apparent. One respondent suggested using the tool during the intake process, thus potentially allowing the client to contribute information directly, emphasizing that this would help practitioners quickly understand the key social relationships in a client’s life. Another respondent expanded on this idea by emphasizing the importance of keeping the network up to date. As relationships evolve (e.g., new individuals enter the network, relationships change, some individuals become less influential), revising the network over time would ensure that it remains a relevant and valuable resource. This theme of maintaining a “living” network was supported across respondents. Another insight was that the tool helps center surface knowledge that practitioners may already possess but have not consciously focused on. By laying out the relationships visually, certain dynamics that were previously in the background come into sharper focus, helping practitioners identify where to direct their attention. Finally, a respondent noted the tool’s utility in both familiar and unfamiliar contexts. For a client whose network is well-known to the supervising officer, the visualization reaffirms that understanding. Conversely, for clients whose networks are less familiar, the tool would help identify knowledge gaps and inform the next steps.

Theme 2: “Value of Multiple Perspectives”. This was gleaned from responses to the following question: “*By asking several of you to provide input, we received multiple perspectives on the client’s social network. We created a “composite” network that shows information gleaned from all the respondent feedback. In reviewing that network, do you see value in having these different perspectives and aggregating them together?*” Participants emphasized the value of incorporating multiple viewpoints. The composite network (i.e., Figure 5 and Figure 7 discussed above) provided a way to highlight not only areas of shared understanding but also gaps in knowledge between respondents. This was especially meaningful in cases where individuals working with the client had varying degrees of insight into different parts of the client’s social world. Specifically, the probation officers had information on offending, while the CBO had information on family relationships. These differences point to the distinctive knowledge held by officers who have insights into connections that may be very important (positively or negatively) to the client but unknown to others involved. All three respondents reported value in collecting and pooling this disparate knowledge. The CBO representative shared that seeing the probation officer’s view of the network alongside their own helped to identify blind spots and opportunities for collaboration. It also enabled them to consider other individuals within the network who could be engaged to support the client. Overall, respondents emphasized that the composite visualization could serve as a tool for integrating different perspectives and identifying new avenues for intervention.

Theme 3: “Communication and Collaboration”. This came from the following question: “*Does this information provide you with a useful tool for communicating with other agencies or groups?*” All three respondents noted that the data collected and the visualization tool would be of great value in facilitating inter-agency communication. As mentioned previously, the CBO representative found it helpful to see the network from the probation officer’s perspective, and this benefit was seen as generalizable to other agency partners. The tool was described as a useful visual aid for discussions involving multi-agency collaborations. It also has potential for use with other stakeholders, including police representatives. By providing a shared reference point, the respondents expressed the belief that the network visualization would support clearer communication and facilitate coordinated efforts across agencies.

Theme 4: “Future Considerations: Network Data Collection Tool”. This theme emerged organically. As the focus group session continued, a recurrent theme mentioned by respondents was that any tool that may be considered would need to be simple, usable, and integrated if it were to hold value for the probation agency. To move away from the physical data collection step used in the pilot, the lead researcher developed a prototype “Network Data Collection Tool” (available for preview at: https://jacobtnyoung.shinyapps.io/example-dashboard/, accessed on 27 January 2026) that delivers the steps developed in the pilot in an electronic format. The respondent enters the names and tie types for important alters. As the respondent does so, the tool generates an image of the network for the respondent to review. As with the pilot, in the second step of the process, the respondent constructs the alter–alter relationships. All respondents emphasized a need to collect data efficiently and dynamically. The prototype tool shown above is a step toward incorporating this insight, as it reduces the burden of physically drawing the networks and allows the respondent to update the network based on new information.

## 4. Discussion

This study described an exploratory pilot project seeking to examine the effectiveness of adapting the group audit method from gang violence research to client supervision by a probation agency. The current study builds on work using group audits by incorporating insights from research on cognitive social structures to measure convergence (as well as divergence) of perceptions. While the group audit method has been used to help design network interventions in law enforcement contexts, this pilot is one of the first to systematically adapt such methods for use in youth probation. In short, the pilot showed that examining belief networks among criminal justice practitioners may be a promising approach for designing network interventions in alternative criminal justice contexts.

The practical benefits of network-informed supervision are considerable. Network data can illuminate hidden influences in a client’s life, both risk-enhancing and protective, that may otherwise go unrecognized. This information has the potential to shape supervision strategies in several ways: prioritizing contacts for engagement, identifying opportunities for targeted interventions, and enhancing multi-agency collaboration by visualizing who is in the client’s orbit and how they are connected. In service delivery terms, this approach could be especially useful for tailoring case plans, allocating resources, or flagging clients who may benefit from more intensive support. Such a tool could be useful to practitioners working directly with clients to facilitate thinking about their network and suggesting ways they can alter their own networks to improve outcomes (e.g., D. P. Kennedy et al., 2022). In complex cases involving individuals at high risk of recidivism and risk of serious harm to others, a network approach can support coordinated assessment and enable effective multi-agency collaboration and monitoring. Such an approach may be especially beneficial in such cases, as it may result in the identification of influential or powerful individuals in the network who might facilitate intervention (e.g., Schiffer & Hauck, 2010).

While the group audit method has been limited to the study of gang violence, the results of this pilot project suggests that the basic insight of the method (that there is valuable knowledge among practitioners and community members for informing interventions) extends beyond gangs: “Such audits need not be restricted to law enforcement experts; instead, the audit process can (and should) be carried out with social service providers, street workers, teachers, and other experts who can speak to the tangle of gang members’ overlapping relationships” (Sierra-Arevalo & Papachristos, 2017, p. 383). The value of experiential assets is not only particularly important for probation officers working with youth who are chronically involved in crime, but also for outreach workers in the community.

This study also speaks to several broader strands of research for future work. First, it overlaps with a substantial body of research on juvenile probation and supervision that highlights the need for individualized case planning, risk assessment, and responsivity to clients’ social environments. The findings further resonate with scholarship on multi-agency coordination, where fragmented knowledge often hinders effective collaboration across justice- and community-based organizations. By making these differences in knowledge explicit, the client network audit approach may offer a mechanism for improving shared situational awareness. In addition, the study contributes to ongoing discussions about the implementation and adoption of decision-support tools in criminal justice settings. Finally, the results raise important questions about the validity and reliability of network elicitation in institutional settings, suggesting that variation across respondents is not merely a measurement error but may reflect meaningful constructs that can be leveraged to improve intervention design.

Positioning the group audit method alongside research on cognitive social structures also provides future avenues of research. As suggested by McCarty (2002), structural properties of ego-centric networks may be of interest when comparing multiple respondents. Future work employing an audit such as the one discussed here may further benefit from descriptive analyses of the structure of the network beyond the current analyses (e.g., modularity analysis) and inferential models such as multi-layer exponential random graph models (see Krivitsky et al., 2020).

While the current findings are promising, it should be kept in mind that this was only a pilot study with a single client. Whether the approach would benefit a larger number of clients is yet to be determined; however, the findings discussed in this study suggest that an approach including multiple actors to address the needs of a single client may benefit from sharing knowledge. Future work should examine whether there is a benefit in extending the approach to more clients. Furthermore, application in other criminal justice settings is an open question for which more research is needed.

Any potential future use would require thoughtful integration into existing practices. While the method is not overly complex, it does require training, modest system adaptations, and agreement on how and when network data should inform decision-making. To mitigate any disruption during the early stages, one option would be to conduct a “live trial” where the network approach is piloted alongside standard supervision processes. This parallel implementation would allow for direct comparison without posing risks to clients or case outcomes. The live trial would also allow practitioners to assess the added value of network data collection and analysis in real time, further informing decision makers in terms of any potential wider roll-out. In addition, the use of a focus group in this pilot project revealed important information about implementation. Future work should consider employing a similar strategy alongside the group audit.

## Figures and Tables

**Table 1:** Edge Types Percentages for Respondent Diagrams.

Edge Type Percentages in Respondent Diagrams			
*Edge Type*	R1 Net (*L* = 87)	R2 Net (*L* = 82)	R3 Net (*L* = 97)
Family	35.63	9.76	45.36
Cooffender	11.49	9.76	0.00
Acquaintance/Friend	40.23	68.29	49.48
Agency	12.64	12.2	5.15

## Data Availability

Data is unavailable due to privacy restrictions.
